# BAD Dephosphorylation and Decreased Expression of MCL-1 Induce Rapid Apoptosis in Prostate Cancer Cells

**DOI:** 10.1371/journal.pone.0074561

**Published:** 2013-09-05

**Authors:** Dana Yancey, Kyle C. Nelson, Daniele Baiz, Sazzad Hassan, Anabel Flores, Ashok Pullikuth, Yelena Karpova, Linara Axanova, Victoria Moore, Guangchao Sui, George Kulik

**Affiliations:** 1 Department of Cancer Biology, Wake Forest School of Medicine, Winston-Salem, North Carolina, United States of America; 2 Department of Chemistry, Elon University, Elon, North Carolina, United States of America; University College London, United Kingdom

## Abstract

PTEN loss and constitutive activation of the PI3K signaling pathway have been associated with advanced androgen-independent prostate cancer. PTEN-deficient prostate cancer C42Luc cells survive in serum-free media and show relative resistance to apoptosis even in the presence of the PI3K inhibitor ZSTK474. Yet, when ZSTK474 is combined with the translation inhibitor cycloheximide, C42Luc cells undergo apoptosis within 6 hours. We identified dephosphorylation of BAD (Bcl2-associated death promoter) as a main apoptosis-regulatory molecule downstream from PI3K, and loss of MCL-1 (Myeloid cell leukemia -1) as a major target of cycloheximide. The combination of MCL-1 knockdown and expression of phosphorylation-deficient mutant BAD2SA is sufficient to trigger rapid apoptosis in prostate cancer cells. These results establish the mechanism for the synergistic induction of apoptosis by the combination of a PI3K inhibitor and of a protein synthesis inhibitor in PTEN-deficient prostate cancer cells.

## Background

Several studies have identified the phosphatidylinositol 3-kinase (PI3K) pathway as one of the major factors in prostate carcinogenesis and progression to therapeutic resistance [1]–[3]. PI3K serves as a mediator of intracellular signal transduction by generating phosphatidylinositol (3–5)-triphosphate (PIP3) through phosphorylation of phosphatidylinositol (4,5)-biphosphate (PIP2). Once generated, PIP3 recruits proteins containing the pleckstrin homology (PH) domain (including AKT kinase) to the cellular membrane, where they undergo a conformational change. In Akt, this conformational change results in a priming phosphorylation at threonine 308 by phosphoinositide-dependent kinase 1 (PDK1) followed by an activating phosphorylation at serine 473 by mammalian target of rapamycin complex 2 (mTORC2) [4]. Activated Akt translocates to the cytoplasm and nucleus to phosphorylate a number of downstream targets involved in cell survival, growth, proliferation, and cell cycle progression[5]. The lipid phosphatase and tumor suppressor PTEN (phosphatase and tensin homolog deleted on chromosome 10) serves as a negative regulator of Akt and the PI3K pathway by dephosphorylating PIP3 and converting it back to PIP2. In prostate cancer, the primary mechanism for PI3K dysregulation is the loss of function of PTEN through homozygous deletions, loss of heterozygosity, or inactivating mutations [6], [7], leading to the constitutive activation of Akt.

Androgen ablation induces apoptosis in prostate epithelial cells [8]. Yet PTEN-negative prostate cancer cells do not undergo apoptosis in the absence of androgens [9]. Similarly, mice with prostate-restricted PTEN knockout have reduced levels of apoptosis and diminished prostate involution upon castration [10]. These results suggest that constitutive activation of the PI3K pathway in PTEN-null advanced prostate tumors contributes to androgen independence by inhibiting apoptosis.

Proteins of the BCL-2 family play a central role in apoptosis by regulating mitochondrial outer membrane permeabilization (MOMP) and the release of apoptosis-inducing proteins such as cytochrome c, SMAC, and apoptosis-inducing factor (AIF) sequestered within the mitochondria [11]. The BCL-2 protein family is divided into three groups based on functionality and presence of conserved BCL-2 homology (BH1-4) domains: multidomain anti-apoptotic proteins, multidomain pro-apoptotic proteins, and BH3-only proteins. Interactions between these groups of the BCL-2 proteins dictate whether a cell lives or dies. Multi-domain anti-apoptotic proteins such as BCL-2, BCL-XL, and MCL-1 prevent MOMP by interacting with and sequestering the multidomain pro-apoptotic Bcl proteins BAK and BAX [12]. BAK and BAX have BH1-3 domains that allow for oligomerization at the mitochondrial outer membrane and subsequent MOMP through pore formation [13]. The BH3-only proteins, such as BAD, NOXA, and PUMA [14]–[16], competitively bind and neutralize anti-apoptotic proteins, allowing BAX/BAK oligomerization and promoting cell death, whereas Bid and Bim can also interact with and activate BAX and BAK, facilitating membrane insertion and MOMP[11].

BH3-only proteins of the BCL-2 family function as sentinels that regulate apoptosis and survival in response to extracellular stimuli through binding to the hydrophobic groove of their anti-apoptotic partners. Each BH3-only protein has a unique profile of binding partners. Thus, BAD has been shown to bind to and neutralize BCL-2, BCL-XL, and BCL-W [14], [17], displacing BAK and BAX and promoting pore formation. However, other anti-apoptotic proteins such as MCL-1 and A1 are not neutralized by BAD, but instead are bound and neutralized by NOXA and PUMA, respectively [15], [17], [18].

Previously, we demonstrated that increased BAD expression promotes prostate cancer cell proliferation [19]. At the same time, BAD phosphorylation status plays a major role in apoptosis regulation by serving as a convergence point of several anti-apoptotic signaling pathways, including constitutively active PI3K [20]. BAD phosphorylation at serines 112 and 136 (based on mouse sequence) [21], [22] facilitates interaction with 14-3-3 chaperones, whereas phosphorylation at S155 within the BH3 domain disrupts binding to BCL-XL or BCL-2 [23]. As a result, phosphorylation inactivates the pro-apoptotic function of BAD by preventing interaction with BCL-2 and BCL-XL. These earlier results suggested that PI3K inhibition and subsequent BAD dephosphorylation would trigger apoptosis in PTEN-negative prostate cancer cells. However, we found that despite rapid BAD dephosphorylation, PI3K inhibition with ZSTK474 induces apoptosis in C42Luc prostate cancer cells at relatively late time points (between 12–24 hours).

We discovered that MCL-1 expression confers resistance to treatments with PI3K inhibitors and BAD dephosphorylation. Conversely, the combination of MCL-1 loss (induced by cycloheximide or shRNA knockdown) and BAD dephosphorylation triggers rapid apoptosis. These results identify BAD and MCL-1 as the main sentinels of pro-apoptotic signaling in the PTEN-deficient prostate cancer cells studied.

## Results

### The combination of the PI3K inhibitor ZSTK474 and the protein synthesis inhibitor cycloheximide induces rapid apoptosis in C42Luc prostate cancer cells

**Figure 1:**
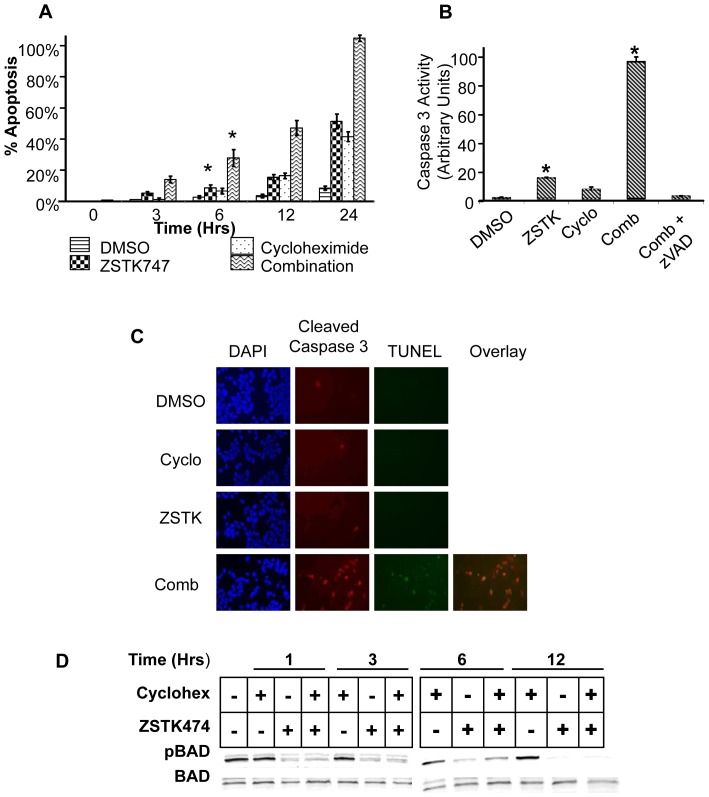
Combination of ZSTK474 and cycloheximide induces rapid apoptosis in C42Luc cells. **A**) C42Luc cells were treated with either 5 µM ZSTK474, 100 µg/mL cycloheximide, or the combination, and apoptosis recorded for 24 hrs using time-lapse microscopy. The cumulative percentage of cells entering apoptosis (rounding and membrane blebbing), is shown at specific time points over 24 h. At least 100 cells were counted for each treatment. Error bars show standard deviations from the average of four randomly chosen fields. *, *p*<0.05. **B**) Caspase-3 activation assay in C42Luc cells treated with 5 µM ZSTK474, 100 µg/mL cycloheximide, or the combination. After 6 h of treatment, cells were lysed, and caspase-3 activity in cell lysates was measured with a fluorogenic substrate (DEVD-AFC). Data are presented as fold-induction of fluorescence intensity normalized to the control (DMSO). *, *p*<0.0001. **C**) Immunofluorescence staining of cleaved caspase 3 (red) and TUNEL (green) in C42Luc cells treated with DMSO, 5 µM ZSTK474, 100 µg/mL cycloheximide, or the combination. DAPI was used to visualize nuclei. Each row of panels represents the same field of view. **D**) Western blot of C42Luc cells treated with either 5 µM ZSTK474, 100 µg/mL cycloheximide, or the combination. Whole cell lysates were collected at the indicated times and probed for pBAD (Ser112) and total BAD.

The PI3K signaling pathway is constitutively activated in C42Luc prostate cancer cells due to a deletion of one allele of the lipid phosphatase PTEN and a frameshift mutation in the other allele [24]. As a result, these prostate cancer cells are not dependent on androgen or other external survival factors. When the PI3K pathway is inhibited by ZSTK474, C42Luc cells undergo apoptosis, as evident from monitoring cells with time-lapse microscopy (Figure 1A, checkered bars).

Comparison of the apoptosis dynamics in C42Luc cells treated with the PI3K inhibitor ZSTK474 alone and in combination with the translation inhibitor cycloheximide has shown that the combination induces apoptosis more rapidly, with a substantial number of cells dying after 6 hours. After treatment with ZSTK474 or cycloheximide alone, apoptosis only became apparent at 12 hours, with a substantial number of cells dying 24 hours after treatments (Figure 1A). Induction of apoptosis by the combination treatment was confirmed by measuring caspase activity with the fluorogenic substrate DEVD-amc, detection of the cleaved active form of caspase 3, and TUNEL assays (Figure 1B, C).

Earlier publications from several laboratories, including ours, showed that phosphorylation of the BH3-only protein BAD plays a central role in apoptosis regulation downstream of the PI3K signaling pathway in various cell lines, including the prostate cancer cell lines LNCaP and C42 [20], [25], [26]. However, BAD dephosphorylation did not differ between cells treated with ZSTK474 alone or with the combination of ZSTK474 and cycloheximide (Figure 1D). These results suggest that BAD dephosphorylation is either irrelevant or insufficient for rapid induction of apoptosis in C42Luc prostate cancer cells.

### Loss of MCL-1 sensitizes prostate cancer cells to apoptosis induced by the PI3K inhibitor ZSTK474

To elucidate the mechanism of the rapid apoptosis induced by cycloheximide in combination with ZSTK474, we turned our attention to other BCL-2 family proteins. Since dephosphorylated BAD can bind BCL-XL, BCL-2, and BCL-W, but cannot interact with MCL-1, we hypothesized that MCL-1 could be responsible for delayed apoptosis in cells with dephosphorylated BAD. Another line of evidence to support a role of MCL-1 in apoptosis regulation is its relatively short half-life, which permits dynamic regulation of MCL-1 levels by extracellular stimuli [27] and makes MCL-1 especially sensitive to inhibition of translation by cycloheximide.

**Figure 2:**
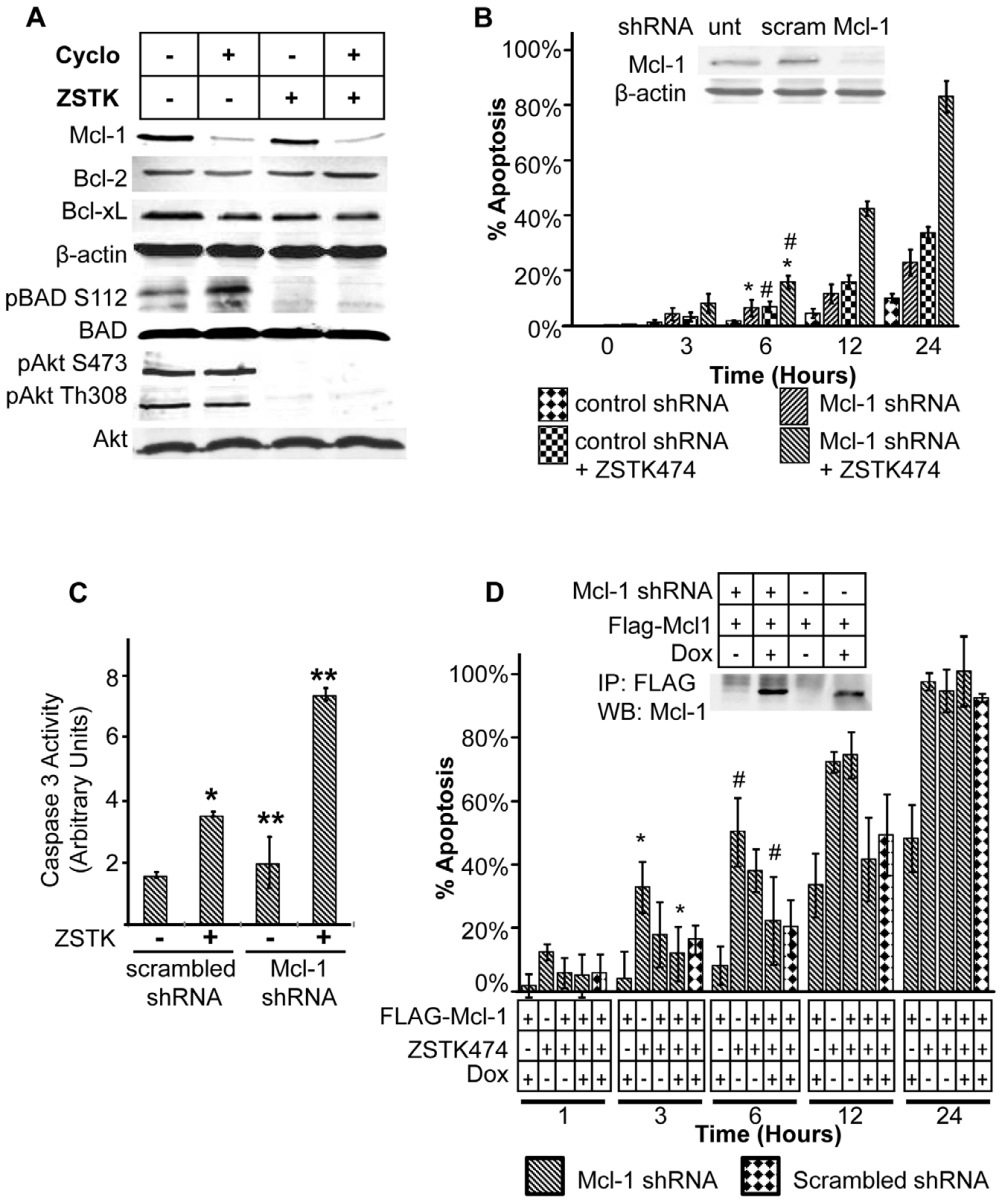
Loss of MCL-1 sensitizes prostate cancer cells to ZSTK474-induced apoptosis. **A**) Western blot analysis of C42Luc cells treated with either 5 µM ZSTK474, 100 µg/mL cycloheximide, or the combination for 6 hours. Whole cell lysates were probed for MCL-1, BCL-2, and BCL-XL to compare the effects of the protein synthesis inhibitor and probed for pBAD (S112) and pAKT (S473 and T308) to verify the effects of the PI3K inhibitor. **B**) Analysis of apoptosis by time-lapse microscopy. C42Luc cells were transiently transfected with lentiviral vector that encodes GFP and MCL-1-specific shRNA or scrambled control shRNA, and treated with 5 µM ZSTK474 48 h after transfection. The percentage of apoptotic cells at indicated time points after treatments was determined as in Figure 1A. At least 100 cells were counted for each treatment. Error bars show standard deviations from the average of four randomly chosen fields. *, *p*<0.005; #, *p*<0.001 Inset shows Western blot of endogenous MCL-1 and β-actin (loading control) levels in C42Luc cells 48 hours after infection with lentiviral vector expressing MCL-1 specific or scrambled shRNA. **C**) Caspase-3 activation assay of C42Luc cells transfected with shRNA targeting MCL-1 or scrambled control shRNA. Caspase activation was measured 6 hours after cells were treated with either DMSO or 5 µM ZSTK474. Treatments were added 48 hours after transfection. *, *p*<0.01; **, *p*<0.05. **D**) Analysis of apoptosis by time-lapse microscopy in C42Luc cells transfected with MCL-1-specific shRNA (striped bars) or scrambled shRNA (checkered bars) and doxycycline-inducible Flag-MCL-1. The cumulative percentage of apoptotic cells at indicated time points was determined as in Figure 1A 24 hours after doxycycline treatment. At least 100 cells were counted for each treatment. Error bars show standard deviations from the average of four randomly chosen fields. *, *p*<0.01 #, *p*<0.05. Inset shows Western blot of Flag-tagged MCL-1 immunoprecipitated with anti-FLAG resin and probed for MCL-1 expression.

Prostate cancer cells commonly express three anti-apoptotic Bcl proteins: BCL-XL, BCL-2, and MCL-1 [28], [29]. Analysis of anti-apoptotic Bcl2 family protein expression levels in C42Luc cells treated with pro-apoptotic agents showed that MCL-1 expression is substantially decreased in cells treated with cycloheximide for 6 hours, and in cells treated with the combination of ZSTK474 and cycloheximide (Figure 2A). In contrast, no significant reductions in BCL-2 and BCL-XL expression were detected in cells treated with a combination of inhibitors. Still, this correlative evidence leaves open the possibility that other short-lived proteins may contribute to increased apoptosis in cells treated with ZSTK474 and cycloheximide.

To directly test the role of MCL-1 as the primary mediator of cycloheximide-induced apoptosis in C42Luc cells, we decreased MCL-1 protein expression by using shRNA knockdown. Analysis of apoptosis by caspase assay and time-lapse microscopy in cells infected with a lentiviral vector that expresses either Mcl1-specific shRNA or scrambled control shRNA showed that ZSTK474 induced apoptosis more efficiently in cells with knockdown of MCL-1 (Figure 2B, C). Conversely, ectopic expression of a doxycycline-inducible Flag-MCL-1 construct decreased apoptosis in cells transfected with MCL-1 shRNA to comparable levels as those in cells transfected with scrambled shRNA (Figure 2D). Increased apoptosis was also observed in ZSTK474 treated cells in which MCL-1 expression was knocked down using a second MCL-1-specific shRNA construct that targeted 3′-UTR (Figure S1). Taken together, the results of these experiments suggest that MCL-1 loss contributes to cycloheximide-induced sensitization to apoptosis induced by ZSTK474.

### BIM is involved in apoptosis regulation in C42Luc cells

**Figure 3:**
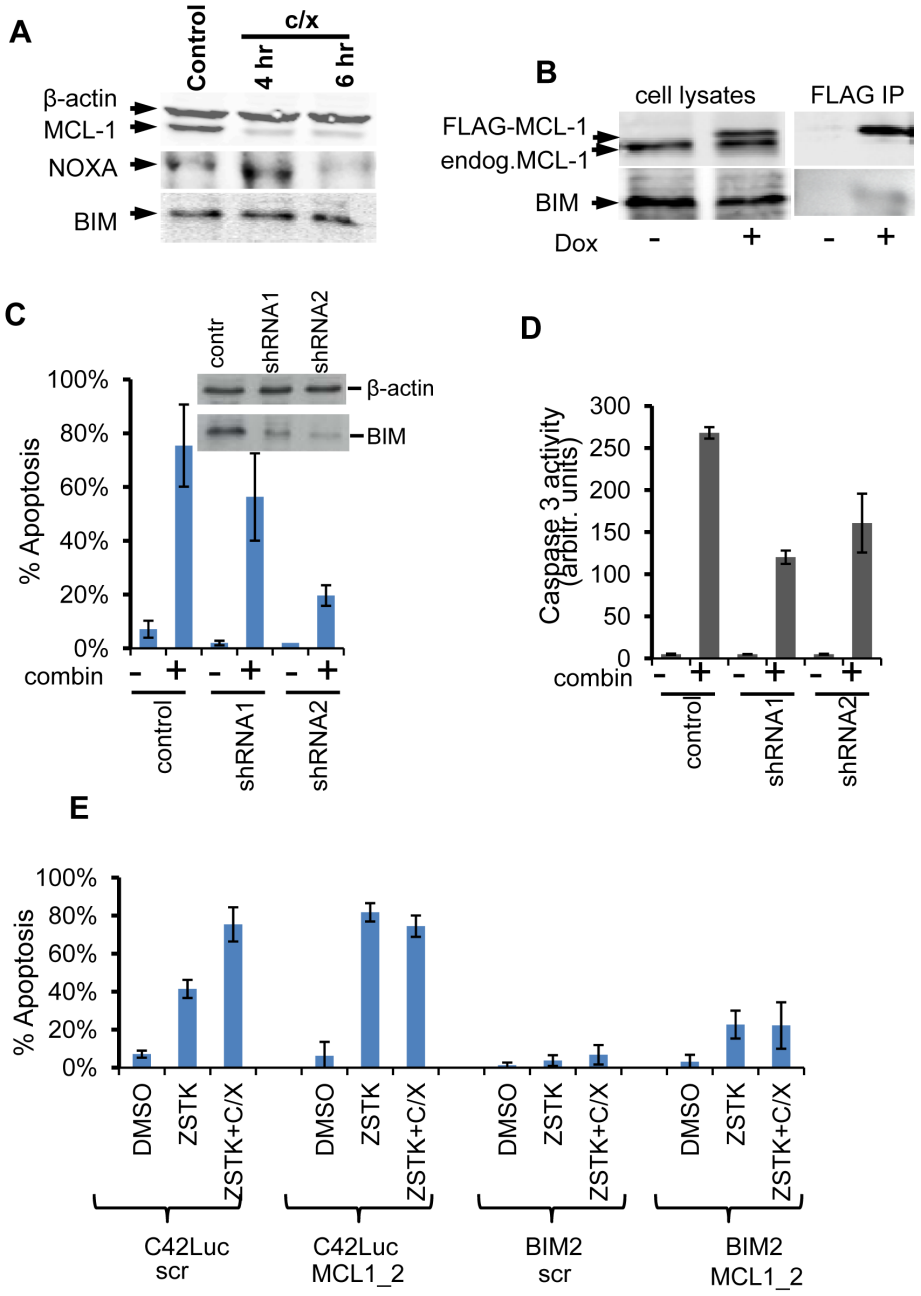
Bim knockdown decreased sensitivity of C42Luc cells to apoptosis. **A**) NOXA expression is decreased in C42Luc cells treated with the combination of ZSTK474 and cycloheximide. Western blot analysis of MCL-1, NOXA, BIM and β-actin (loading control) in cells treated for 4 and 6 hours with cycloheximide. **B**) Co-immunoprecipitation of MCL-1 and BIM from cells that expressed doxycycline-inducible FLAG-MCL-1. **C**) Apoptosis in C42Luc cells that express two BIM-specific shRNA and in parental cells was assessed by time lapse microscopy. Cells were treated with vehicle ((–) DMSO) or combination of cycloheximide and ZSTK474 (+). Amount of apoptosis (%) was determined as in Figure 1A. At least 100 cells were counted for each treatment. Error bars show standard deviations from the average of four randomly chosen fields. Data for 6 hours post-treatment are presented. Differences in% apoptosis between control cells (that express scrambled shRNA) and C42Luc BIM1 or C42Luc BIM2 cells were statistically significant (p<0.05 and p<0.0001, respectively). Insert shows decreased BIM expression in cells that stably expressed BIM-specific shRNA. **D**) Apoptosis in C42Luc cells that expressed one of two BIM-specific shRNA or control vector was assessed by measuring caspase activity. Cells were treated for 6 hours with vehicle ((–) DMSO) or the combination of cycloheximide and ZSTK474 (+). Differences in caspase activity between control cells and C42Luc BIM1 or C42Luc BIM2 cells were statistically significant (p = 0.002 and p = 0.05, respectively). **E**) C42Luc cells or C42LucBIM2 cells that stably express BIM-targeting shRNA were transfected with lentiviral vector that express GFP and either scrambled (scr) or MCL-1 –specific shRNA2. Percent of apoptosis in GFP-positive cells was determined by time-lapse microscopy. At least 100 cells were counted for each treatment. Error bars show average and standard error of four visual fields. Qualitatively similar results were obtained in C42LucBIM1 cells. Beta actin was used as a loading control in (A) and (C).

BIM and NOXA, BH3-only proteins of the Bcl2 family, have been identified as binding partners of MCL-1. NOXA binds predominantly to MCL1, whereas BIM can also bind other anti-apoptotic proteins of the Bcl2 family, as well as bind to and activate BAX [15], [30]. NOXA, but not BIM, is reportedly involved in the protein complex that targets MCL-1 for degradation [31]. Indeed, in C42Luc cells treated with cycloheximide, NOXA was degraded whereas BIM expression remained constant (Fig. 3A). Thus, we focused on the analysis of BIM. Consistent with earlier reports, BIM was detected in immunoprecipitates with FLAG beads from C42Luc cells that express FLAG-MCL-1 (Fig. 3B). To test whether BIM is involved in apoptosis induction by the combination of cycloheximide and ZSTK474, we used two BIM-specific shRNAs to knock down expression of endogenous BIM protein. Significant reduction of caspase activity, as well as decreased apoptosis in both C42Luc-shBIM1 and C42Luc-shBIM2 cells compared to control C42Luc cells, was detected after treatments with cycloheximide and ZSTK474 (Fig. 3C, D), confirming the role of BIM in apoptosis of C42Luc cells. Furthermore, decreased apoptosis was observed in both C42Luc-shBIM1 and C42Luc-shBIM2 cells compared with C42Luc cells transfected with a construct that expressed MCL-1-specific shRNA, indicating that BIM is involved in apoptosis initiation “downstream” of MCL-1 loss.

### BAD dephosphorylation is responsible for the rapid apoptosis induced by the PI3K inhibitor ZSTK474 plus cycloheximide

**Figure 4:**
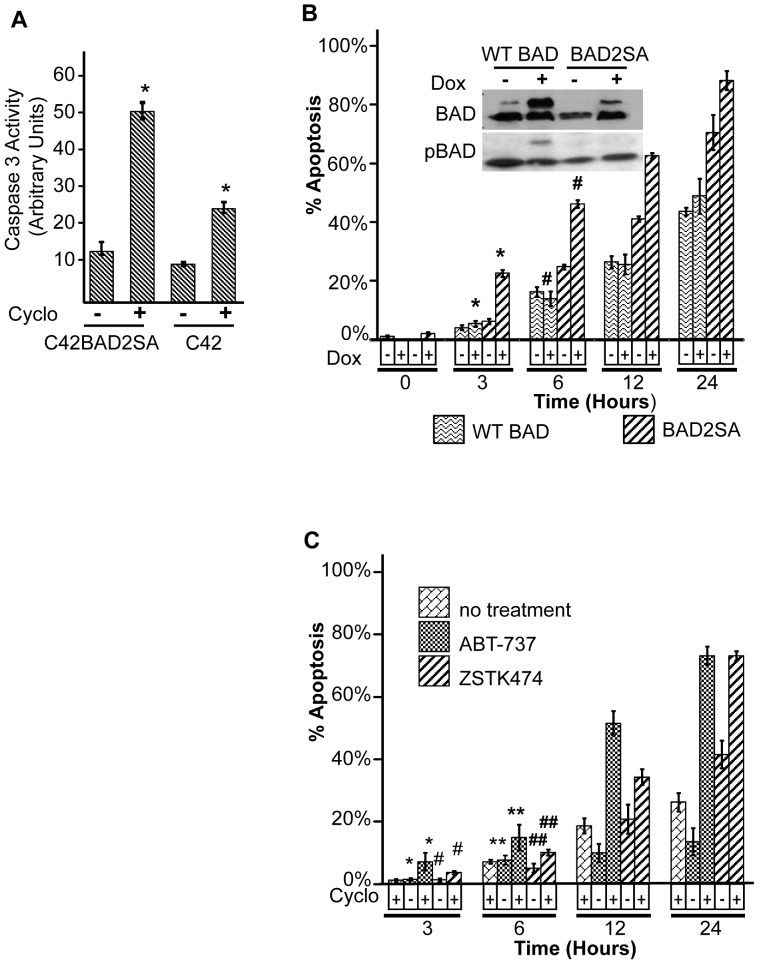
Phosphorylation-deficient BAD and a BAD mimetic sensitizes prostate cancer cells to apoptosis by protein synthesis inhibitors. **A**) Caspase 3 activation in C42Luc and C42LucBAD2SA cells that expressed a phosphorylation-deficient BAD mutant. Cells were treated with 100 µg/mL cycloheximide for 6 hours before assay. *, *p*<0.01. **B**) Analysis of apoptosis by time-lapse microscopy. C42Luc cells were transiently transfected with a doxycycline-inducible HA-BAD2SA or wild-type HA-BAD, and treated with doxycycline (Dox) 48 hours after transfection. After 18 h of Dox treatment, cells were treated with 100 µg/mL cycloheximide, and the cumulative percentage of apoptotic cells was determined by time lapse microscopy. At least 100 cells were counted for each treatment. Error bars show standard deviations from the average of four randomly chosen fields. *, *p*<0.01 #, *p*<0.03. Inset shows Western blot analysis of phosphorylated BAD (pBAD112) and total BAD expression in C42Luc cells collected 18 hours after Dox treatment. Lower bands correspond to endogenous protein, while the upper bands correspond to inducibly expressed HA-BAD constructs. **C**) C42Luc cells were treated with 100 µg/ml cycloheximide in combination with 10 µM ABT-737, a BH3 mimetic based on the BH domain of BAD or ZSTK474. Apoptosis was analyzed by time-lapse microscopy. Cumulative cell death at indicated time points is shown. At least 100 cells were counted for each treatment. Error bars show standard deviations from the average of four randomly chosen fields. *, *p*<0.01 **, *p*<0.05 #, *p*<0.001 ##, *p*<0.002.

Earlier, we demonstrated that treatment with a PI3K inhibitor triggers BAD dephosphorylation at S112 and S136; we also used shRNA knockdown to demonstrate that BAD is necessary for apoptosis induction by PI3K inhibitors in prostate cancer cells [20]. Still, several targets of PI3K signaling besides BAD were implicated in inhibition of apoptosis [32]. To determine whether BAD dephosphorylation is sufficient to induce rapid apoptosis in the presence of cycloheximide, C42Luc cells were transfected with a doxycycline-inducible phosphorylation-deficient mutant BAD, where Ser112 and Ser136 were substituted for alanines (BAD2SA). Upon cycloheximide treatment, cells transfected with BAD2SA demonstrated significantly increased apoptosis compared to cells transfected with wild-type BAD (WT-BAD) (Figure 4A and B). Consistent with a central role of BAD in apoptosis regulation, treatment of prostate cells with a BAD-mimetic ABT-737 (based on the BH3 domain of BAD) induced increased apoptosis when combined with cycloheximide, similar to the combination of cycloheximide with the PI3K inhibitor ZSTK474 (Figure 4C).

### Simultaneous BAD dephosphorylation and MCL-1 loss is sufficient to induce rapid apoptosis in C42 cells

**Figure 5:**
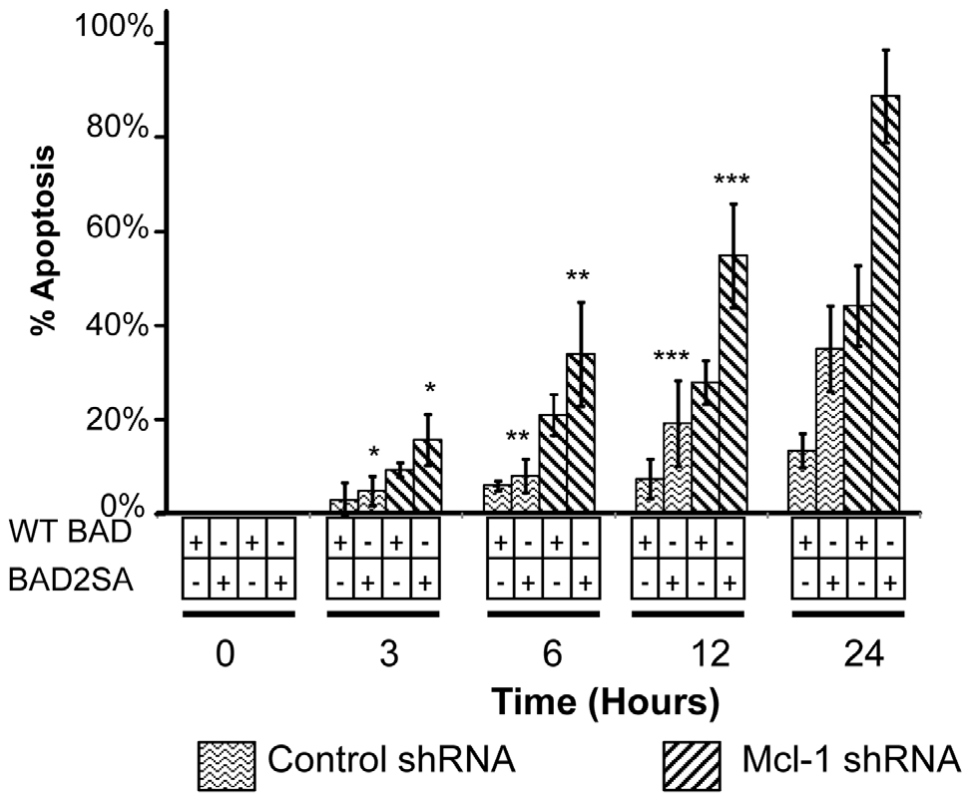
Knockdown of MCL-1 and ectopic expression of phosphorylation-deficient BAD are sufficient to induce apoptosis in prostate cancer cells. C42Luc cells were co-transfected with either MCL-1-specific shRNA or scrambled shRNA in combination with either the BAD2SA or WT-BAD construct. 48 hours post-transfection, apoptosis was analyzed by time-lapse microscopy. At least 100 cells were counted for each treatment. Error bars show standard deviations from the average of four randomly chosen fields. *, *p*<0.05 **, *p*<0.005 ***, *p*<0.001.

To confirm that BAD dephosphorylation and MCL-1 loss are sufficient to induce rapid apoptosis, C42Luc cells were co-transfected with BAD2SA and MCL-1shRNA, and apoptosis was evaluated by time-lapse microscopy. Cells co-transfected with BAD2SA and MCL-1 shRNA showed significantly more apoptosis compared to cells transfected with the combination of WT-BAD and MCL-1 shRNA or BAD2SA and scrambled shRNA (Figure 5). These results identify BAD and MCL-1 as key players in regulating apoptosis in C42Luc prostate cancer cells.

**Figure 6:**
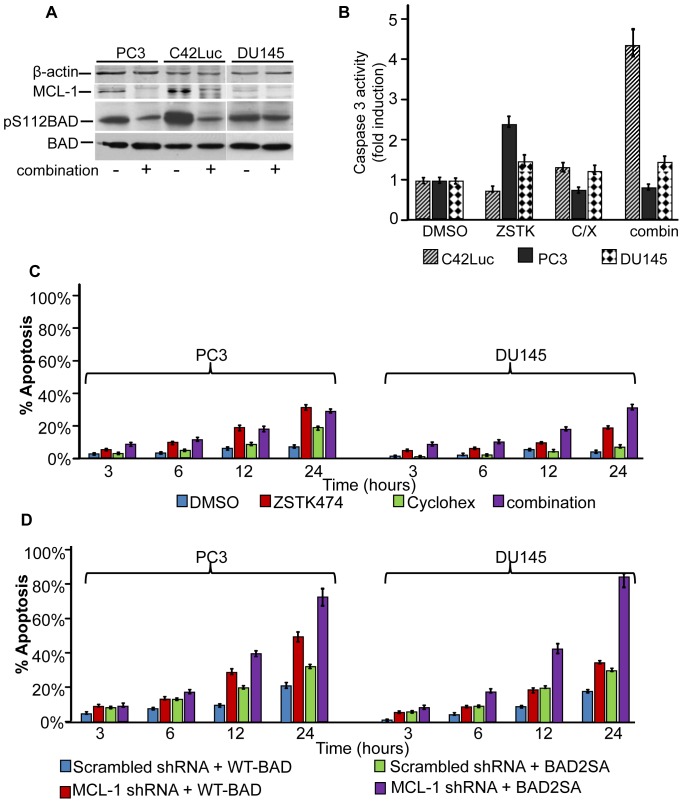
DU145 and PC3 cells are less sensitive to apoptosis than C42Luc cells. **A**) Treatment with combination of cycloheximide and ZSTK474 triggers MCL-1 loss in PC3, C42Luc, and DU145 cells, as well as BAD dephosphorylation in PTEN-deficient PC3 and C42Luc cells. Cell lysates prepared 6 hours after treatments were probed for pS112BAD, total BAD, and MCL-1, using β-actin as a loading control. Apoptosis in C42Luc, PC3, and DU145 cells was assessed by (**B**) measuring caspase 3 activity with fluorogenic substrate or (**C**) by time lapse microscopy. At least 100 cells were counted for each treatment. Error bars show standard deviations from the average of four randomly chosen fields. **D**) Knockdown of MCL-1 and ectopic expression of phosphorylation-deficient BAD were sufficient to induce apoptosis in prostate cancer cells. PC3 or DU145 cells were co-transfected with MCL-1-specific shRNA or scrambled control shRNA in combination with either a BAD2SA or WT-BAD expression construct. 48 hours post-transfection, apoptosis was analyzed by time-lapse microscopy. At least 100 cells were counted for each treatment. Error bars show standard deviations from the average of four randomly chosen fields.

To address the generality of apoptosis induction by the combination of cycloheximide and ZSTK474 in prostate cancer cells, we assessed apoptosis in WFU3, PC3, and DU145 cells by time-lapse microscopy and caspase-3 fluorometric assays. PTEN^−/−^WFU3 mouse prostate epithelial cells showed induction of apoptosis comparable to C42Luc cells (Figure S2). In contrast, the induction of apoptosis in PC3 and DU145 cells by combination of cycloheximide and ZSTK474 was substantially less compared to C42Luc cells (compare Figures 1A and 6C), despite the dephosphorylation of BAD and loss of MCL-1 expression in treated cells (Figure 6A, B, C). Yet when these cell lines were co-transfected with expression vectors encoding MCL-1 shRNA and BAD2SA with mutated phosphorylation sites, substantial induction of apoptosis was observed in all cell lines (Figure 6D, Figure 5). This result led us to hypothesize that different sensitivity to apoptosis in cell lines may depend on expression profiles of Bcl2 family in these cells.

**Figure 7:**
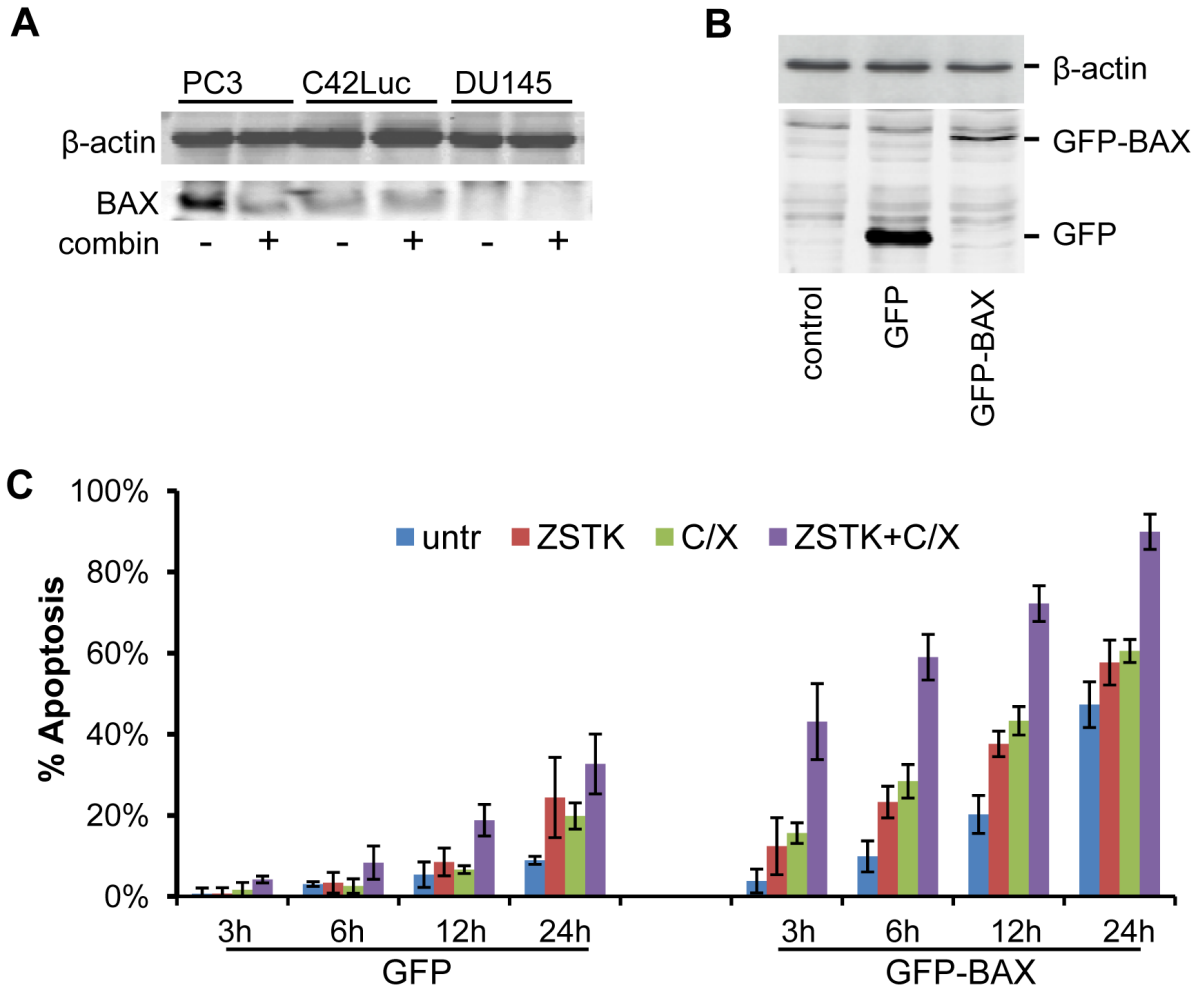
Expression of BAX sensitizes DU145 cells to apoptosis. **A**) Western blot analysis of BAX expression in PC3, C42Luc, and DU145 cells. Cell lysates prepared 6 hours after treatments were probed with antibodies against BAX and β-actin. **B**) Western blot analysis of cells transfected with green fluorescent protein (GFP) or GFP-BAX chimera. **C**) Expression of GFP-BAX accelerates apoptosis in DU145 cells. Cells were transfected with GFP or GFP-BAX and apoptosis was analyzed by time-lapse microscopy. At least 100 cells were counted for each treatment. Error bars show standard deviations from the average of four randomly chosen fields.

Indeed, it has been previously reported that PC3 cells express higher levels of BCL-XL, whereas DU145 cells show decreased expression of BAX (Fig. 7A) [33], [34]. Since it has been demonstrated that knockdown of BCL-XL sensitizes cells to apoptosis [35], we focused on testing the role of BAX expression in apoptotic response of DU145 cells. DU145 cells were transfected with GFP-BAX or GFP expression vectors, and apoptosis in GFP-positive cells was followed by time-lapse microscopy. DU145 cells transfected with GFP-BAX showed increased apoptosis when treated with the combination of ZSTK474 and cycloheximide. Thus, BAX expression restores sensitivity of DU145 prostate cancer cells to apoptosis induced by combination treatments (Figure 7).

## Discussion

### Mechanistic understanding of the induction of apoptosis is necessary to improve efficacy of PI3K inhibitors

Dysregulation of the PI3K pathway has been well documented in a variety of cancer types, with approximately 40% altered PI3K signaling at primary prostate tumor sites [36], [37] and 100% altered signaling at metastatic sites [38]. In prostate cancer, the primary mechanisms for PI3K dysregulation are loss of function of PTEN through homozygous deletions, loss of heterozygosity, or inactivating mutations, leading to the constitutive activation of PI3K/Akt signaling. The functional loss of PTEN is associated with advanced cancer progression, high Gleason grade, and overall poor prognosis, underlining the need for chemotherapeutic options for tumors with active PI3K/Akt signaling [39]–[41].

Several small molecule inhibitors of the PI3K pathway, including the pan-PI3K inhibitors ZSTK474 (Zenyaku Kogyo Co) and BKM120 (Novartis), and the dual PI3K/mTOR inhibitor BEZ235 (Novartis) have already entered early-phase clinical trials for treatment of solid malignancies, including prostate cancer [42]–[44]. However, earlier trials did not report significant improvements in clinical outcomes [45], [46].

A better understanding of the mechanisms of cancer cell resistance to PI3K inhibition is needed to select optimal therapeutic regimes for tumors with active PI3K signaling. Specifically, information about proteins that control apoptosis in PTEN-deficient prostate cancer cells would guide the selection of agents to increase sensitivity of prostate cancer cells to apoptosis.

### BAD and MCL-1 are critical apoptosis-regulatory molecules in prostate cancer cells

Our earlier work described a signaling network of convergent signaling pathways that control BAD phosphorylation and thus, apoptosis in prostate cancer cells [20]. We also showed that BAD knockdown makes PTEN-deficient prostate cancer LNCaP cells insensitive to apoptosis induced by PI3K inhibitors. These earlier data, together with results presented here showing increased apoptosis in cells that express phosphorylation-deficient mutant BAD2SA, suggest that BAD is a critical regulator of apoptosis targeted by PI3K signaling. Thus, BAD phosphorylation appears to be a useful biomarker to predict the anti-tumor efficacy of PI3K inhibitors. Analysis of BAD phosphorylation is particularly relevant considering that other signaling pathways activated by receptor tyrosine kinases and G-protein coupled receptors can phosphorylate BAD even when the PI3K pathway is inactive. Thus, despite Akt dephosphorylation (routinely used as a marker of PI3K pathway activity), cells may not undergo apoptosis if BAD is phosphorylated by other signaling pathways.

Our data demonstrate that BAD dephosphorylation in itself is insufficient to induce rapid apoptosis in prostate cancer cells. Analysis of the mechanisms of rapid apoptosis induced by the combination of PI3K inhibitors and translation inhibitors identified MCL-1 as a critical apoptosis-regulatory molecule. Its expression, when decreased, cooperates with BAD in inducing apoptosis. Both BAD and MCL-1 are ubiquitously expressed in prostate tumors [28] and could be dynamically regulated by phosphorylation [20] (in the case of BAD) and both phosphorylation and ubiquitylation (in the case of MCL-1) [47].

Overexpression of MCL-1 has been associated with advanced prostate cancer, including high Gleason grade primary tumors and metastatic tumors, and hematopoietic malignancies [28], [48]. MCL-1 has been identified as a critical regulator of cell survival, and is subject to multiple levels of regulation. Transcriptionally, MCL-1 mRNA levels are rapidly induced by a number of signal transduction pathways, including the MAP/ERK, PI3K/Akt, and JAK/STAT pathways, with aberrant activation in a variety of malignancies [49]. MCL-1 mRNA is also tightly regulated by mir29 b [50]. MCL-1 protein has a high turnover rate through ubiquitin-dependent protein degradation by the 26 S proteosome. In healthy cells, the BH3 domain of the MULE E3 ligase binds to the hydrophobic groove of MCL-1 specifically and displaces its interaction with BAK and BH3-only sentinels [51]. MCL-1 is phosphorylated by glycogen synthase kinase-3β (GSK3β) in stressed cells where the PI3K/Akt pathway is inactive, and primed for ubiquitination by the E3 ligase β-TrCP [27], [47]. The high turnover rate of MCL-1 permits dynamic regulation of MCL-1 expression at the levels of transcription, translation, and degradation. These multiple levels of regulation highlight the role of MCL-1 as a critical apoptosis regulator and attractive therapeutic target, as well as a potential biomarker.

Earlier, MCL-1 was implicated in anti-apoptotic signaling by IL-6 in LNCaP and Du145 prostate cancer cells, whereas downregulation of MCL-1 lead to apoptosis in two days [52]. Here we report that a combination of MCL-1 loss and BAD dephosphorylation is sufficient to induce rapid apoptosis in PTEN-deficient advanced prostate cancer cells. Also, apoptosis in C42Luc cells was decreased by knocking down expression of BIM (Figure 3), identifying BIM as important regulator of apoptosis downstream of MCL-1 in C42Luc prostate cancer cells.

The process of apoptosis via mitochondrial pathways could be broadly divided into two phases: initiation (pre-MOMP) and execution (post-MOMP). Reports on the dynamics of cytochrome c release and phosphatidylserine externalization, indicated that post-MOMP phase is relatively short (30–60 min) and uniform (all cells that release cytochrome c externalize phosphatidylserine and die). In contrast, the pre-MOMP phase is stochastic, and its average duration is determined by relative levels of BCL-2 family proteins [53], [54]. Thus, either dephosphorylating BAD or decreasing MCL-1 expression alone leads to delayed apoptosis. In contrast, treatments that induce simultaneous BAD dephosphorylation and MCL-1 loss trigger rapid apoptosis in prostate cancer cells. Unlike PI3K inhibitors that relatively are well tolerated, inhibitors of protein synthesis are characterized by general toxicity. Thus, prostate tumor-specific inhibitors of protein synthesis are needed to test if the combination of PI3K and of protein synthesis inhibitors will show antitumor efficacy in animal models of prostate cancer.

PC3 and DU145 cells are less sensitive to apoptosis due to increased expression of BclXL or loss of BAX, respectively. These differences in sensitivity to apoptosis induced by combination therapy underscore the limitation of this study, suggesting that only a subset of advanced prostate tumors is sensitive to combination therapy.

Considering heterogeneity of advanced prostate tumors, a personalized approach in selecting patients who would respond best to combination therapies provides hope to improve therapy outcomes. Our results suggest that comprehensive system analysis of expression levels of BCL2 family proteins in tumors is needed to identify patients who will benefit from therapies that target apoptosis.

## Methods

### Cell culture and treatment

C42Luc cells were generated from C42 cells (gift from Dr. Leland Chung, Cedars-Sinai Medical Center, Los Angeles, California, USA)[55] by stably expressing firefly luciferase (PGL4.13)[19]. PC3 and DU145 cells were obtained from the ATCC. C42shBIM1 and C42shBIM2 stable cell lines that express BIM-specific shRNA were generated from C42Luc cells infected with lentivirus vectors containing BIM-targeted shRNA and a puromycin resistance marker. Cells were grown in RPMI-1640 Complete Cell Culture Media (Gibco) supplemented with 10% fetal bovine serum. PTEN^−/−^WFU3 cultured adult mouse prostatic epithelial cells infected with lentivirus containing PTEN shRNA to develop a PTEN^−/−^ stable cell line [56] were a generous gift from Dr. Scott Cramer (University of Colorado Denver, Dept of Pharmacology). PTEN^−/−^WFU3 cells were grown in complete medium consisting of 50∶50 Dulbecco's modified Eagle's medium (DMEM)/Ham's F-12 medium (F12) supplemented with 10 mg/ml fraction V bovine serum albumin (Sigma-Aldrich), 1% fetal bovine serum, cholera toxin (10 ng/ml; List Biologicals, Campbell, CA), epidermal growth factor (10 ng/ml; BD Biosciences, San Jose, CA), bovine pituitary extract (28 *µ*g/ml; Hammond Cell Tech, Windsor, CA), gentamicin (80 mg/ml; Sigma-Aldrich), insulin (8 *µ*g/ml; Calbiochem, La Jolla, CA), *α*-tocopherol (2.3×10^6^ M), transferrin (5 *µ*g/ml; Sigma-Aldrich), and trace elements (final concentrations in medium: 1 nM MnCl_2_, 500 nM Na_2_SiO_3_, 1 nM (NH_4_)_6_Mo_7_O_24_, 5 nM NH_4_VO_3_, 500 pM NiCl_2_, 50 pM SnCl_2_, 20 nM H_2_SeO_3_). All cells were maintained at 37°C with 5% CO_2_. One hour prior to treatment, all cells were placed in serum-free media before the following treatments (final concentrations) were added for the indicated amount of time: 5 µM ZSK-474, 100 µg/µL of cycloheximide, and 10 µM ABT-737. Combination treatment was the addition of both ZSTK-474 and cycloheximide.

### Plasmids and transfection

C42tet-on cells were transiently transfected with HA-BAD2SA-pTRE-tight (BAD2SA) and HA-BAD-pTRE-tight (WT BAD) or were stably transfected with pcDNA3-HA-BAD (WT BAD) and pcDNA3-HA-BADS112/136A (BAD2SA) constructs (gift from Michael Greenberg, Harvard Medical School, Boston, MA).

Two MCL-1 and BIM targeted-shRNA were generated using the vector-based RNAi technology previously described [57].

Briefly, polymerase chain reaction was used to amplify the U6 promoter region that was digested with BamHI and HindIII restriction sites. Oligonucleotides containing the target sequences (MCL1shRNA1, 5′- AATTCAAAAAATTGTTTAACTCGCCAGTCCCGT A -3′; MCL1shRNA2, 5′-GTAGCCAGGCAAGTCATAGAATA-3′; BIMshRNA1, 5′-GAC CAC CCA CGA ATG GTT ATC TA -3′; BIMshRNA2 5′-GGTTATCTTACGACTGTTACGTA-3′) and a 6 nucleotide hairpin sequence were annealed and digested by HindIII-EcoRI. The pLL3.7 lentiviral vector was digested with BamHI and EcoRI. The U6 promoter and MCL-1 target oligonucleotide were ligated at HindIII, and the remaining digested ends ligated to the digested pLL3.7 vector at BamHI and EcoRI sites. A scrambled RNA oligonucleotide sequence 5′-GGTACGGTCAGGCAGCTTCT-3′ was used as a control. Transfections were performed at 60–70% confluence using Lipofectamine (Invitrogen, Carlsbad, CA) according to the manufacturer's recommendations. Alternatively, C42Luc cells were infected with lentivirus produced in 293 cells as previously described [20]. GFP-fusion wild-type BAX construct was generated using the C3-EGFP plasmid (Clontech Laboratories, Inc., Palo Alto, CA).

### Protein expression

Concentrations of whole cell lysates were determined by Bio-Rad Protein Assay (Bio-Rad). 100 µg protein lysate for whole cells was separated on 10% or 12.5% SDS/PAGE gels, transferred to nitrocellulose membrane, blocked in 3% BSA for one hour, and incubated at 4°C overnight with primary antibody and one hour with secondary antibody at room temperature. Detection was performed using the Amersham ECL™ Western Blot Detection Reagents (GE Healthcare) and Odyssey Fluorescence Imaging. Antibodies used for detection of MCL-1 (1∶3000; Stressgene; CA). p-AKT Thr308 (1∶1000 dilution), p-AKT Ser473 (1∶1000 dilution), total AKT (1∶1000 dilution), BCL-2 (1∶1000 dilution), and BCL-XL (1∶1000 dilution) were obtained from Cell Signaling Technologies (Beverly, MA). β-actin (1∶5000 dilution) was obtained from Sigma (Santa Cruz, CA). Rabbit IgG IRDye800 Goat Polyclonal Conjugated Secondary Antibody and Mouse IgG IRDye680LT Goat Polyclonal Conjugated Secondary Antibody (1∶3000 dilution) were obtained from Li-Cor Biosciences (Lincoln, NE).

### Analysis of apoptosis

#### Time-lapse Microscopy

Cells were plated in 6-well plates (200,000 cells/well) and transfected by Lipofectamine with a mixture of GFP-tagged MCL-1-specific shRNA or scrambled shRNA (control); and constructs of WT BAD or BAD2A in a 1∶1 ratio. After 48 hours, cells were serum starved overnight (16 hours) before treatment with 5 µM ZSTK474, 100 µg/ml cycloheximide, the combination, or DMSO as a control. GFP-positive cells were measured by time-lapse video recording followed by counting the percentage of cells with apoptotic morphology (assessed as cytoplasmic blebbing and fragmentation). For these experiments, at least four randomly chosen fields (containing on average 100–200 cells for each treatment) were recorded. Video recording was performed on an Axiovert100 microscope (Carl Zeiss, Germany) equipped with a moving stage and climate control chamber (37°C, 5% CO_2_) and controlled by Openlab software (Improvision Inc., Lexington, MA). The results reported herein were confirmed by at least three independent experiments.

#### DEVDase Assay

Cells were plated into 6 cm dishes (400,000 cell/plate), serum-starved overnight (16 hours) and treated with 5 µM ZSTK474, 100 µg/mL of cycloheximide, or both. Apoptosis in whole cell populations was quantified by measuring caspase-3 activity with the fluorogenic substrate Ac-DEVD-7-amido-4-trifluoromethyl-coumarin (Bachem) as specified by the manufacturer. For these experiments, attached and floating cells were collected and lysed in caspase lysis buffer (1% Nonidet P-40, 150 mm NaCl, 20 mm HEPES, 1 mm EDTA, 1 mm dithiothreitol, and 5 µg/ml aprotinin, leupeptin, and pepstatin). Fluorescence was recorded every 15 minutes for 1 hour, and caspase activity was expressed in arbitrary units.

#### Double immunofluorescence for cleaved caspase 3 and TUNEL

To visualize the co-localization of activated caspase 3 and DNA fragmentation in prostate cancer cells (C42LucBAD cells), prostate cancer xenografts (C42LucBAD xenografts), and mice prostate glands, sequential immunofluorescence for cleaved caspase3 and TUNEL (3′ terminal deoxynucleotidyl transferase(*T*dT)-mediated dUTP nick end labeling) was done as described previously [58]. Briefly, cells and fresh frozen tissue sections were fixed in 10% buffered formalin for 20 minutes and then permeabilized in 0.1% sodium citrate with 0.1% NP-40 at 4°C for 2 minutes, followed by blocking for 30 minutes with 2.5% goat serum in PBS-t (PBS+0.1% Tween 20) at 37°C. Primary antibody to cleaved caspase 3 (Cell Signaling Technology) (1∶300 in PBS-t with 2.5% goat serum) was added for 3 hours at room temperature. After washing 3 times in PBS-t, secondary antibody conjugated with Texas Red (1∶300 in PBS-t+2.5% goat serum) was added for 90 minutes at room temperature. After washing 3 times in PBS-t, TUNEL reaction was done as described in the manual for the In Situ Cell Death Detection Kit (Hoffmann-La Roche Ltd).

#### Statistical Analysis

To determine whether differences between data sets were statistically significant, Student's *t* test analysis (2-tailed distribution; 2-sample unequal variance) was performed using Microsoft Excel software. For comparison of percent apoptosis nonparametric Mann-Whitney test was calculated using Prism software. A *P* value less than 0.05 was considered significant. Error bars show standard deviations from the average of at least 3 samples.

## Supporting Information

Figure S1 Knockdown of MCL-1 sensitize cells to apoptosis. Analysis of apoptosis by time lapse microscopy. C42Luc cells were transiently transfected with lentiviral vector that encodes GFP and MCL-1shRNA2 or scrambled control shRNA, and treated with 5 µM ZSTK474 (ZSTK) 48 hours after transfection. At least 100 cells were counted for each treatment. Error bars show standard deviation from the average of four counted fields. Treatments with ZSTK474 induced significantly higher apoptosis in cells that express MCL-1 shRNA2 compared to cells that expressed scrambled shRNA (p<0.02). Inset shows Western blot of endogenous MCL-1 and β-actin (loading control) levels in HEK293 cells 48 hours after infection with lentiviral vector expressing MCL-1shRNA2 or in parental C42Luc cells (contr).
(TIF)

Figure S2 Combination of cycloheximide and ZSTK474 induces apoptosis in WFU3 cells. **A**) PTEN^−/−^WFU3 cells were treated with either 5 µM ZSTK474 (ZSTK), 100 µg/mL cycloheximide (cyclo), or the combination, and recorded for 24 hours by time-lapse microscopy. The cumulative percentage of cells entering apoptosis (rounding and membrane blebbing) is shown at specific time points over 24 hours. At least 100 cells were counted for each treatment. Error bars show standard deviations from the average of four randomly chosen fields.**B**) Caspase-3 activation assay of PTEN^−/−^WFU3 cells treated with 5 µM ZSTK474, 100 µg/mL cycloheximide, or the combination. After 6 hours of treatment, cells were lysed, and caspase-3 activity in cell lysates was measured with a fluorogenic substrate (DEVD-AFC). Data are presented as fold-induction of fluorescence intensity normalized to the control (DMSO). Western blot of PTEN^−/−^WFU3 cells treated with either 5 µM ZSTK474, 100 µg/mL cycloheximide, or the combination. Whole cell lysates were collected at 6 hours and probed for pBAD (Ser112), MCL-1, and β-actin.
(TIF)
