# Supplementary figures and images for: BAD Dephosphorylation and Decreased Expression of MCL-1 Induce Rapid Apoptosis in Prostate Cancer Cells
Source: PLoS One. 2013 Sep 5;8(9):e74561. doi: 10.1371/journal.pone.0074561 (PMC3764099; doi:10.1371/journal.pone.0074561)

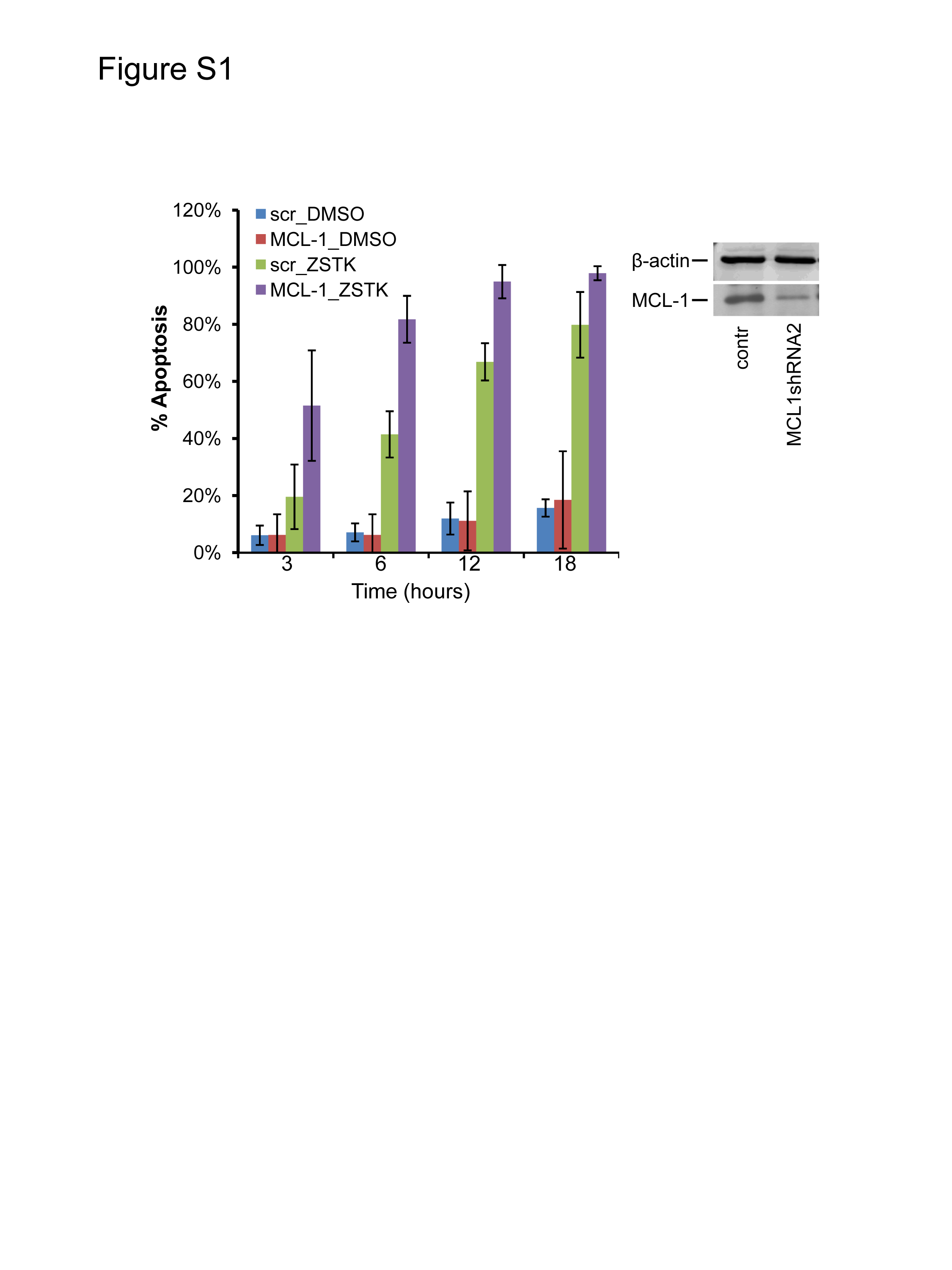

Supplement: Figure S1 — Knockdown of MCL-1 sensitize cells to apoptosis. Analysis of apoptosis by time lapse microscopy. C42Luc cells were transiently transfected with lentiviral vector that encodes GFP and MCL-1shRNA2 or scrambled control shRNA, and treated with 5 µM ZSTK474 (ZSTK) 48 hours after transfection. At least 100 cells were counted for each treatment. Error bars show standard deviation from the average of four counted fields. Treatments with ZSTK474 induced significantly higher apoptosis in cells that express MCL-1 shRNA2 compared to cells that expressed scrambled shRNA (p<0.02). Inset shows Western blot of endogenous MCL-1 and β-actin (loading control) levels in HEK293 cells 48 hours after infection with lentiviral vector expressing MCL-1shRNA2 or in parental C42Luc cells (contr). (TIF) [file pone.0074561.s001.tif]

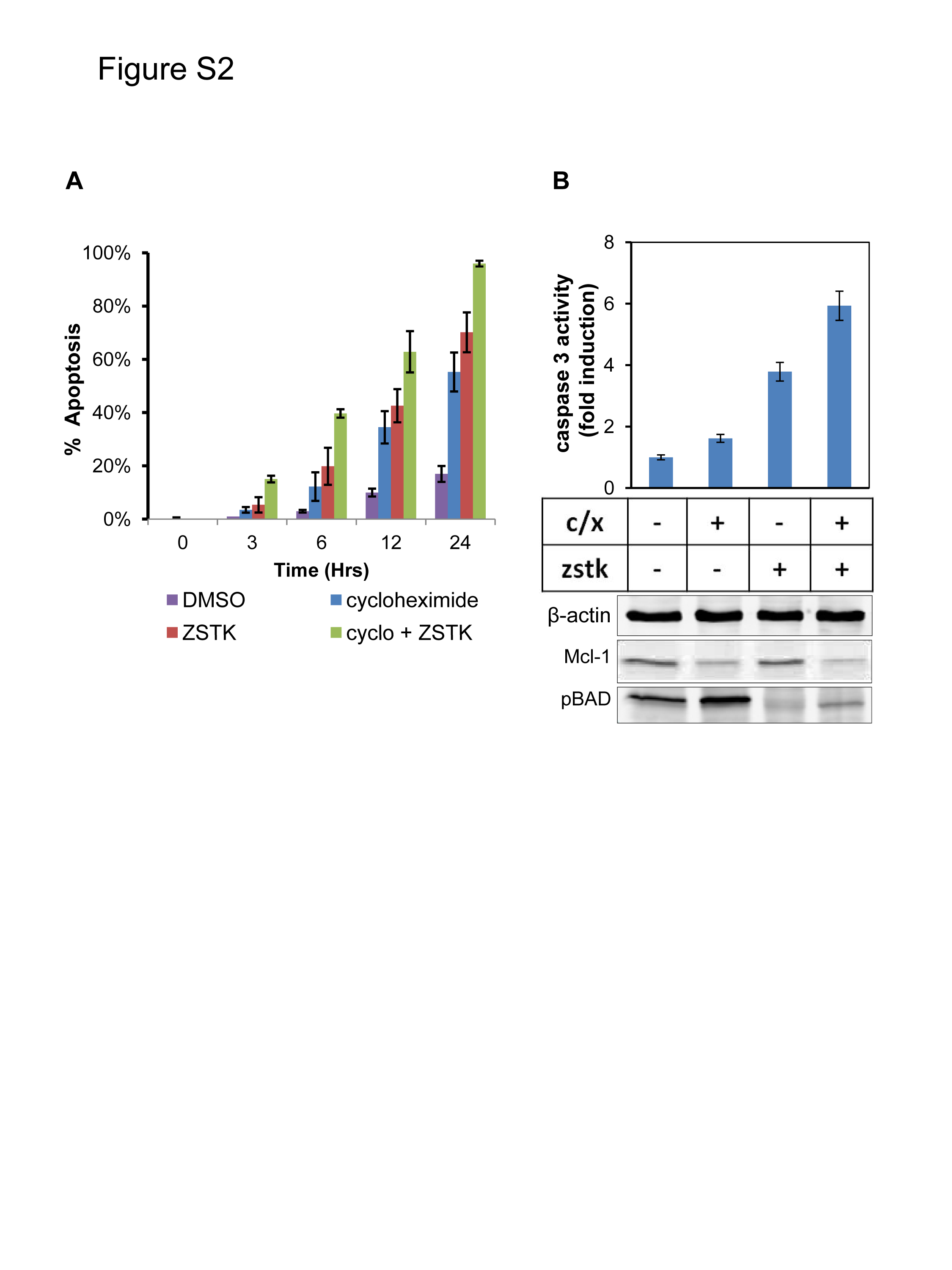

Supplement: Figure S2 — Combination of cycloheximide and ZSTK474 induces apoptosis in WFU3 cells. A) PTEN−/−WFU3 cells were treated with either 5 µM ZSTK474 (ZSTK), 100 µg/mL cycloheximide (cyclo), or the combination, and recorded for 24 hours by time-lapse microscopy. The cumulative percentage of cells entering apoptosis (rounding and membrane blebbing) is shown at specific time points over 24 hours. At least 100 cells were counted for each treatment. Error bars show standard deviations from the average of four randomly chosen fields.B) Caspase-3 activation assay of PTEN−/−WFU3 cells treated with 5 µM ZSTK474, 100 µg/mL cycloheximide, or the combination. After 6 hours of treatment, cells were lysed, and caspase-3 activity in cell lysates was measured with a fluorogenic substrate (DEVD-AFC). Data are presented as fold-induction of fluorescence intensity normalized to the control (DMSO). Western blot of PTEN−/−WFU3 cells treated with either 5 µM ZSTK474, 100 µg/mL cycloheximide, or the combination. Whole cell lysates were collected at 6 hours and probed for pBAD (Ser112), MCL-1, and β-actin. (TIF) [file pone.0074561.s002.tif]
